# Behavioral Outcome Effects of Serious Gaming as an Adjunct to Treatment for Children With Attention-Deficit/Hyperactivity Disorder: A Randomized Controlled Trial

**DOI:** 10.2196/jmir.5173

**Published:** 2016-02-16

**Authors:** Kim CM Bul, Pamela M Kato, Saskia Van der Oord, Marina Danckaerts, Leonie J Vreeke, Annik Willems, Helga JJ van Oers, Ria Van Den Heuvel, Derk Birnie, Thérèse AMJ Van Amelsvoort, Ingmar HA Franken, Athanasios Maras

**Affiliations:** ^1^Yulius AcademyYulius Mental Health Care OrganizationBarendrechtNetherlands; ^2^Department of Clinical PsychologyErasmus University RotterdamRotterdamNetherlands; ^3^Serious Games InstituteCoventry UniversityCoventryUnited Kingdom; ^4^Faculty of Psychology and Educational SciencesKU LeuvenLeuvenBelgium; ^5^Department of Developmental PsychologyUniversity of AmsterdamAmsterdamNetherlands; ^6^Cognitive Science Centre AmsterdamUniversity of AmsterdamAmsterdamNetherlands; ^7^Department of PsychiatryKU LeuvenLeuvenBelgium; ^8^Venture & Incubation CentreJanssen PharmaceuticalsBeerseBelgium; ^9^Department of Medical AffairsJanssen PharmaceuticalsTilburgNetherlands; ^10^Centre ZitStilAntwerpBelgium; ^11^Focuz Treatment Centre for Children and YouthRotterdamNetherlands; ^12^Department of Psychiatry and PsychologyMaastricht UniversityMaastrichtNetherlands; ^13^Mondriaan Mental Health CareHeerlenNetherlands; ^14^Virenze Mental Health CareMaastrichtNetherlands

**Keywords:** attention deficit-hyperactivity disorder, ADHD, serious game, Internet, children, treatment, randomized controlled trial

## Abstract

**Background:**

The need for accessible and motivating treatment approaches within mental health has led to the development of an Internet-based serious game intervention (called “Plan-It Commander”) as an adjunct to treatment as usual for children with attention-deficit/hyperactivity disorder (ADHD).

**Objective:**

The aim was to determine the effects of Plan-It Commander on daily life skills of children with ADHD in a multisite randomized controlled crossover open-label trial.

**Methods:**

Participants (N=170) in this 20-week trial had a diagnosis of ADHD and ranged in age from 8 to 12 years (male: 80.6%, 137/170; female: 19.4%, 33/170). They were randomized to a serious game intervention group (group 1; n=88) or a treatment-as-usual crossover group (group 2; n=82). Participants randomized to group 1 received a serious game intervention in addition to treatment as usual for the first 10 weeks and then received treatment as usual for the next 10 weeks. Participants randomized to group 2 received treatment as usual for the first 10 weeks and crossed over to the serious game intervention in addition to treatment as usual for the subsequent 10 weeks. Primary (parent report) and secondary (parent, teacher, and child self-report) outcome measures were administered at baseline, 10 weeks, and 10-week follow-up.

**Results:**

After 10 weeks, participants in group 1 compared to group 2 achieved significantly greater improvements on the primary outcome of time management skills (parent-reported; *P*=.004) and on secondary outcomes of the social skill of responsibility (parent-reported; *P*=.04), and working memory (parent-reported; *P*=.02). Parents and teachers reported that total social skills improved over time within groups, whereas effects on total social skills and teacher-reported planning/organizing skills were nonsignificant between groups. Within group 1, positive effects were maintained or further improved in the last 10 weeks of the study. Participants in group 2, who played the serious game during the second period of the study (weeks 10 to 20), improved on comparable domains of daily life functioning over time.

**Conclusions:**

Plan-It Commander offers an effective therapeutic approach as an adjunct intervention to traditional therapeutic ADHD approaches that improve functional outcomes in daily life.

**Trial Registration:**

International Standard Randomized Controlled Trial Number (ISRCTN): 62056259; http://www.controlled-trials.com/ISRCTN62056259 (Archived by WebCite at http://www.webcitation.org/6eNsiTDJV).

## Introduction

Attention-deficit/hyperactivity disorder (ADHD) is the most common childhood neurodevelopmental disorder with young patients experiencing functional impairments in different areas of daily life [1-5]. Compared to children without the disorder, children with ADHD have more difficulties at school making schedules to finish assignments on time, executing complex planning tasks, organizing material needed for assignments, remembering task instructions, and setting priorities [6,7]. Thus, it is not surprising that children with ADHD are more likely to show academic underachievement, poor academic performance, and educational problems compared to their counterparts without the diagnosis [8]. Children with ADHD also show impairments in social functioning. They are rejected more often by their peers and have more conflicts with other children and adults compared to their counterparts who do not have ADHD [9]. Although understudied, impaired social functioning in children with ADHD has serious long-term consequences for the development of conduct disorder and even some substance use disorders [10]. Without proper interventions, functional impairments in the areas of time management, planning/organizing, and prosocial behavior skills often endure and escalate into adolescence and adulthood [6,7,11-14].

Although stimulant medication has been shown to reduce ADHD core symptoms among children with ADHD, effects are limited with regard to children’s behavioral, social, and cognitive functioning in daily life [15]. Behavioral interventions developed to improve these children’s functional outcomes, although effective [7,14,16], are often time-consuming, costly, and not easily accessible to all children who might benefit from them [17-19]. Moreover, it appears that 50% of patients with ADHD discontinue treatment regardless of its efficacy or symptom severity [20]. Because of their difficulties with sustaining attention and motivation, patients with ADHD experience low engagement during therapy [21]. Consequently, there is a need to explore more rich interactive experiences with visual effects in computer-based therapy approaches in addition to traditional pharmacological, school-based, and mental health approaches that positively impact the daily life functioning of children with ADHD. The use of Internet-based therapeutic approaches to support and improve health care is growing because of their potential to offer attractive, easily accessible, and efficient interventions outside the clinical setting [19,22,23]. This fits into the World Health Organization Mental Health Action Plan 2013-2020, which promotes accessible user-driven options emphasizing early intervention and autonomy of individuals, thereby promoting nonpharmacological therapies for young patients [22].

A growing number of computerized training programs for ADHD have been designed to improve working memory and executive functioning, thereby addressing specific neurocognitive deficits and ADHD core symptoms [24,25]. Commercial versions of the tasks used in these studies have become readily available (eg, Cogmed, Cognifit, and Memory Booster) [26]. Although these programs show some evidence for short-term effects on targeted working memory outcomes as measured by neurocognitive tests similar to the ones practiced in the games, they have not shown compelling evidence that these effects generalize beyond neurocognitive outcomes to important domains of functioning in the daily lives of children with ADHD (so-called “far-transfer effects”) [27-29]. These findings are consistent with studies examining the effectiveness of “brain training” games within a “normal” population [30]. Moreover, few have game mechanics features with a narrative journey structure. It is worth exploring whether or not an Internet-based therapeutic approach with richer interactive experiences with visual effects could improve functional outcomes in children with ADHD.

Serious gaming (ie, [digital] games used for purposes other than purely entertainment) is a novel and promising approach to support the treatment of clinical symptoms and improvement of adaptive functioning among diverse patient groups [31-35]. Such games offer an environment in which attractive learning tasks are presented in a way that addresses the difficulties that children with ADHD often have in engaging with “boring” and repetitive training tasks [36-38]. These games are characterized by a high-intensity immediate reinforcement and this appears to improve task performance, especially within ADHD populations [39,40]. Serious games differ from existing computerized neurocognitive training programs in several ways. Firstly, they offer an overall game environment that allows for exploration and a meaningful ongoing “journey narrative” instead of offering a “casual” gamelike interface [41]. Secondly, these games not only focus on repeating training tasks, but also offer behavioral strategies (eg, reinforcement, immediate performance feedback from a mentor, goal setting through missions, modeling, social support, and comparison) to increase daily life functioning, thereby potentially enhancing generalization effects. Serious games offer an attractive and accessible online learning environment in which children with ADHD stay motivated to train their skills and learn strategies to deal with impairments that affect functional outcomes in daily life. Although the scientific evaluation of serious games precludes making conclusive statements about their impact on “real-world” behaviors, several controlled trials of serious games have shown to affect these behaviors in diverse patient groups [42].

To our knowledge, a serious game designed to enhance behavior strategies for children with ADHD to improve their daily life functioning has not been scientifically evaluated in the literature. We developed a serious game intervention for children with ADHD to teach and reinforce daily life skills, such as time management, planning/organizing, and cooperation skills [43]. Previous exploratory research in a pilot study of a prototype of the game demonstrated improvement of time management (KCMB, unpublished data, 2016). This study examines the effects of this serious game (called “Plan-It Commander”) as an additional Internet-based adjunct to the treatment of ADHD in children. We hypothesized that participants playing the serious game would improve on primary outcome measures of time management, planning/organizing, and cooperation skills compared to participants in the crossover control group. We hypothesized that participants would also improve on secondary outcome measures of working memory, social skills (ie, responsibility, assertiveness, and self-control) and self-efficacy because these skills were also trained within the overall game environment. We further hypothesized that treatment effects would be maintained at 10-week follow-up for the group that played the serious game for the first 10 weeks of the study.

## Methods

### Participants

A total of 182 participants were recruited from January to March 2013 across 4 outpatient mental health care clinics and institutions in the Netherlands and Belgium. Eligible parents and children were informed by their clinician about this study. In other cases, the patient organization provided information about the study to their members; these parents directly applied for the study. Once the clinician identified eligible parents and children, they received detailed written and verbal information about the study from the researcher. After signing informed consent, they were invited for a screening visit (performed by trained research assistants with MA in psychology) to verify inclusion and exclusion criteria. This resulted in a sample of 170 participants. Inclusion criteria were (1) a *Diagnostic and Statistical Manual of Mental Disorders, Fourth Edition, Text Revision* (*DSM-IV-TR*) diagnosis of ADHD (confirmed by the Kiddie Schedule for Affective Disorders and Schizophrenia-Lifetime version [K-SADS] [44,45]), (2) aged between 8 and 12 years, (3) stable on pharmacological and/or psychological treatment for ADHD 8 weeks before baseline (determined by health care professionals on the basis of medication data and behavioral observation), (4) no initiation or change of pharmacological and/or psychological treatment for ADHD during the study period, (5) availability of a computer workstation at home with Internet and sound facilities, and (6) sufficient understanding of the Dutch language by the child and by at least one of the parents. ADHD severity was measured by the parent version of the Disruptive Behavior Disorder Rating Scale (DBDRS) [46,47] and children with common comorbid disorders of ADHD (eg, oppositional defiant disorder as measured by the DBDRS) could participate in the study. Exclusion criteria were (1) an estimated total Intelligent Quotient (IQ) lower than 80 (determined by vocabulary and block design subtests of the Wechsler Intelligence Scale for Children III [WISC-III] [36,48,49], (2) substance abuse problems (eg, drugs, alcohol), (3) conduct disorder, (4) autism spectrum disorder (both previously diagnosed by health care professionals), (5) comorbid acute psychiatric disorder (eg, depression, mania; confirmed by the K-SADS [44,45]), and (6) participation in a previous pilot study with a prototype of Plan-It Commander. Children with a severe physical disability (eg, blindness, deafness) or learning disability (eg, dyslexia) were also excluded on the basis of the child’s medical file and a standardized interview administered by phone to parents. Written informed consent was obtained from parents and children aged 12 years. All study procedures were approved in advance by the Erasmus (Dutch; MEC-2012-539) and Leuven (Belgian) Medical Ethical Committees.

### Design

**Figure 1:**
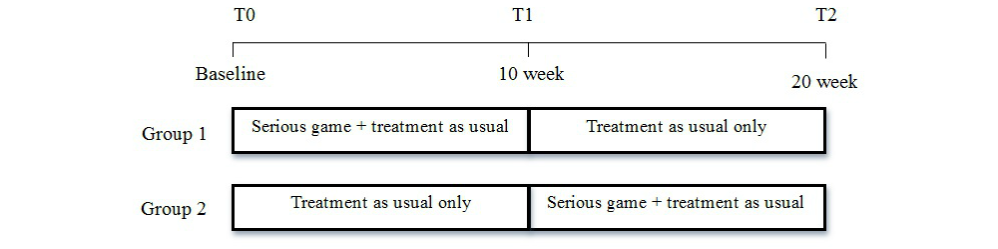
Study design with both groups.

This study used a 20-week multisite randomized controlled crossover open-label trial design (see Figure 1). The intervention was an online serious game called Plan-It Commander. Participants were randomized to a serious game intervention group (group 1) or a treatment-as-usual crossover group (group 2). Participants randomized to group 1 received a serious game intervention in addition to treatment as usual for the first 10 weeks and then received treatment as usual only for the next 10 weeks. Participants randomized to group 2 received treatment as usual for the first 10 weeks and crossed over to the serious game intervention in addition to treatment as usual for the subsequent 10 weeks. All participants in the study received treatment as usual and most participants (91.8%, 156/170) were on medication. Participants were instructed to play the serious game for a maximum of 65 minutes approximately 3 times per week. The game was programmed so that participants could not play more than 65 minutes in one 24-hour period to prevent excessive use of the game. The CONSORT EHEALTH checklist is presented as Multimedia Appendix 1

### Randomization and Blinding

Randomization was carried out on a 1:1 ratio and based on a prespecified computer-generated randomization list. Allocation was stratified by study site and gender and arranged in permutated blocks. Group assignment was performed online using the next available number on the randomization list corresponding to the site and gender of the participant. It was not possible to blind participants to their treatment allocation. After screening and baseline assessment, parents received an email with the notification to which group (group 1 vs group 2) their child was allocated. Although all efforts were made to keep the investigator blind during baseline assessments, full blinding of researchers and teachers at the other assessment points could not be guaranteed because participants could spontaneously talk about the game during the study.

### Intervention

The serious game is an online adventure game (called Plan-It Commander) developed by health care professionals, researchers, and game experts in collaboration with parents and children with ADHD. In collaboration with a focus group of parents, the multidisciplinary game development team agreed on the game’s learning goals and play frequency/time. After each prototype build, usability tests were iteratively performed to examine whether children liked the game and understood how to use it and navigate within the game. User data were evaluated and incorporated in the design process for the final game format, which was examined in this study. Plan-It Commander was designed to improve domains of daily life functioning with a primary focus on time management, planning/organizing, and cooperation skills in children with ADHD. Unifying their knowledge and expertise resulted in a unique online learning environment in which principles of behavior therapy and game-based learning were combined [43]. Players had their own password and ID to log on to the Internet-based serious game from their home where they could access 2 game components: (1) a mission-guided game environment with minigames related to the learning goals of time management, planning/organizing, and cooperation skills and (2) a closed social community. The game was linked to a database in which data about play frequency and duration were registered from each participant.

**Figure 2:**
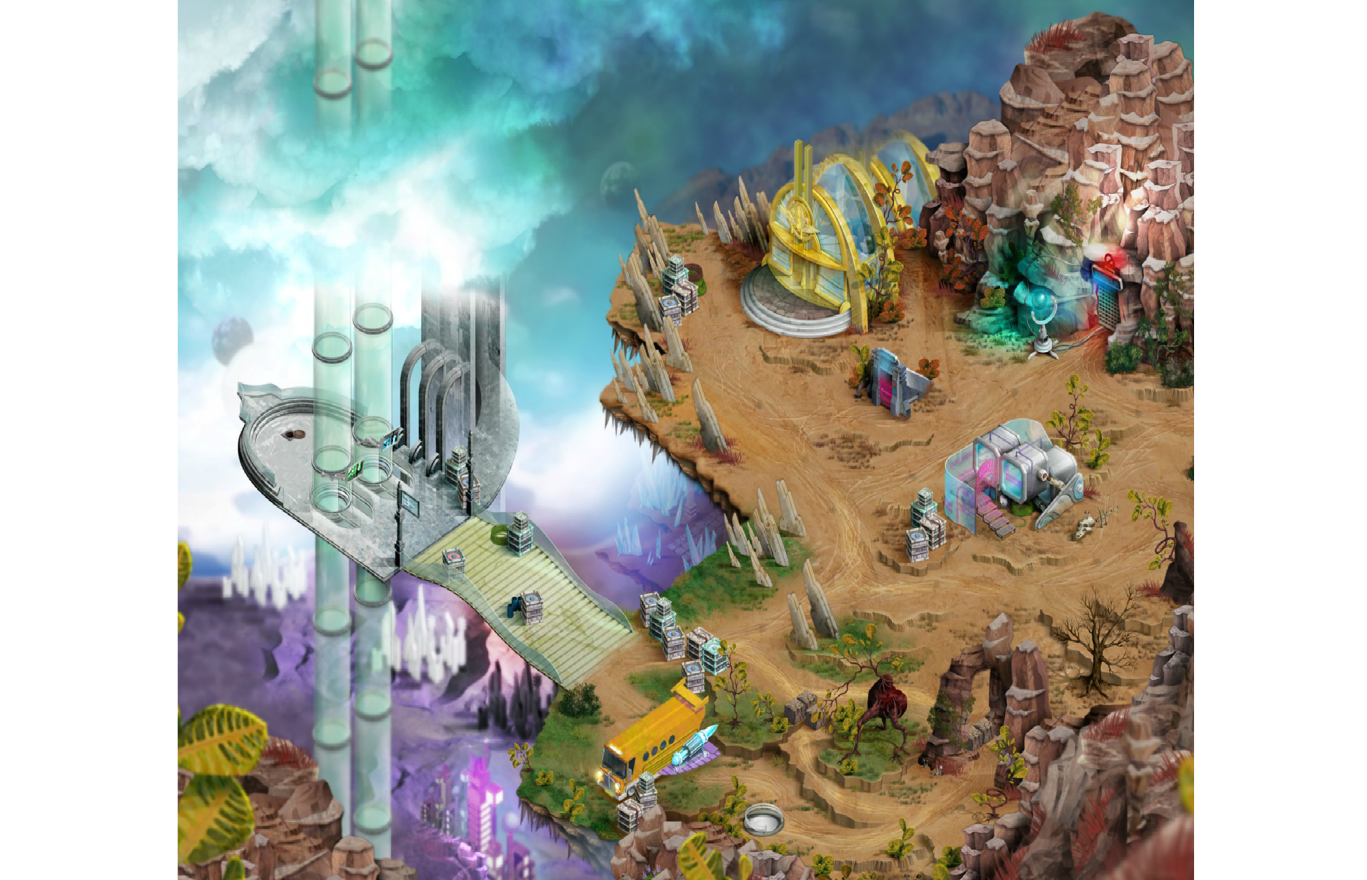
Screenshot of Plan-It Commander game world.

**Figure 3:**
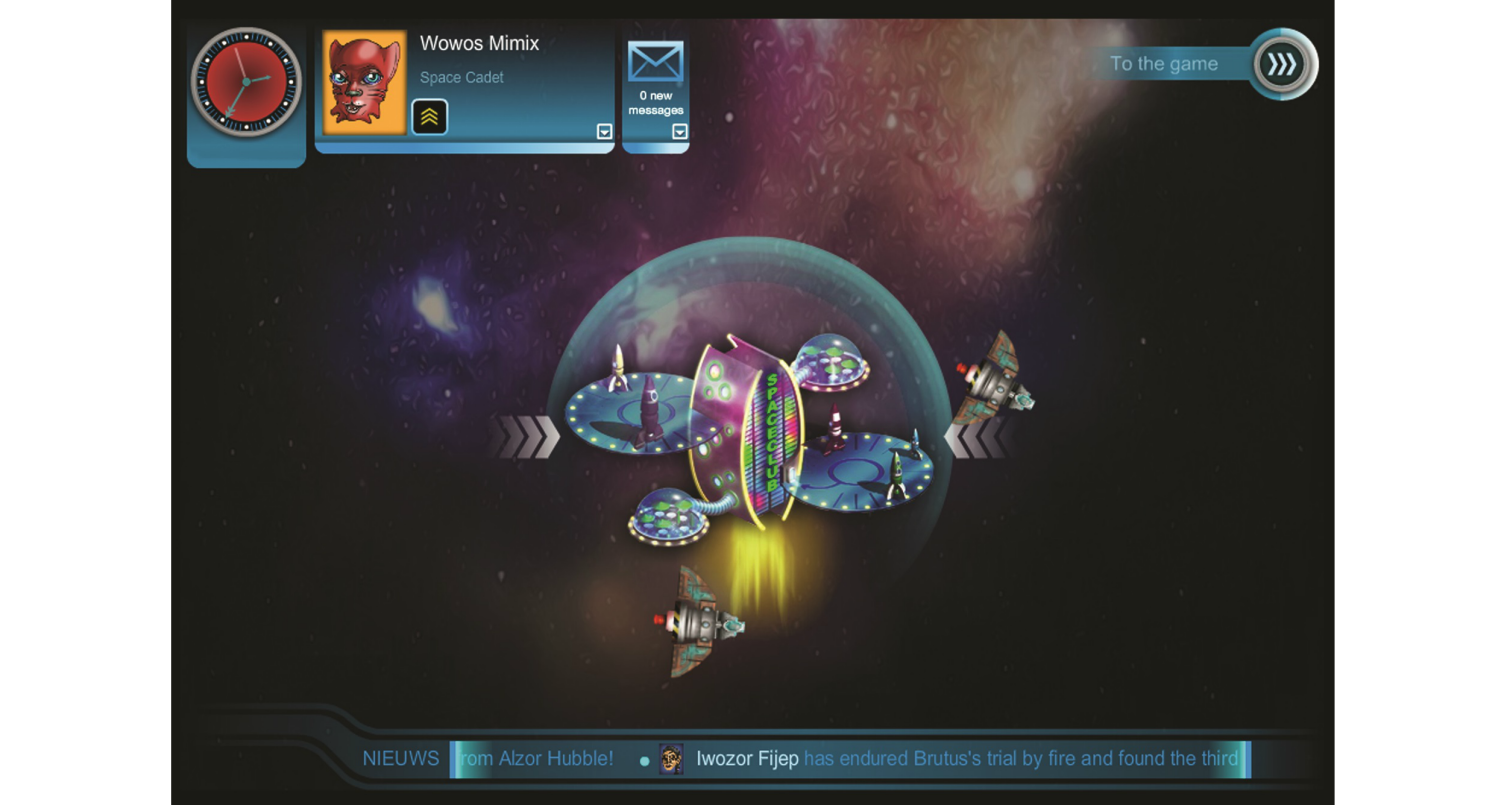
Screenshot of game social community (called “Space Club”).

Plan-It Commander is a mission-guided game divided into 10 different missions and several side missions (Figure 2). Missions guide the player’s behavior throughout the game as he or she follows the storyline and is asked to solve problems requiring specific skills. Central parts are the 3 minigames addressing time management, planning/organizing, and prosocial behavior that are embedded in the structure of the game. The first minigame is focused on teaching the player time estimation and time management skills. The second minigame is focused on enhancing planning skills; the player is taught to plan ahead and break down the total assignment into pieces. The third minigame focuses on enhancing prosocial behavior, teaching the player to help their team members and to cooperate with each other. In addition to the mission-guided game, players could access a closed social community (called “Space Club”) to stimulate prosocial behavior (eg, helping other players, giving compliments) (Figure 3). Players can ask for help or help other players through predefined messages and reward them with a thank you message. The player’s profile is presented within the community and shows an overview of his or her progression throughout the game. When a player completes certain “challenges” in the mission-guided game, an achievement is unlocked in the community. Every player has an overview of awarded achievements in the form of badges or medals in their profile within the community. By making progress in the game and reaching certain milestones, the player unlocks rewards in the community. Rewards can vary in form, such as papercraft models, desktop wallpapers, and music from the game. Players can see each other’s profiles and this generates competition between players. Details of the development and content of Plan-It Commander are described elsewhere [43].

### Measures

Multi-informant (parent, teacher, and self-report) measures were administered at baseline (T0), at 10 weeks (T1), and at 10-week follow-up (T2). Parent and teacher reports were administered through online questionnaires. Questionnaires were administered to the children during face-to-face appointments at each assessment time point. At baseline, demographic information and children’s game experience were collected through parent reports. The parent reported on the game experience of their child as starter, amateur, experienced, or expert. For the primary outcomes, parents filled in the following questionnaires during the 3 assessment time points: (1) a time management questionnaire (Multimedia Appendix 2), (2) the subscale Plan/Organize of the Behavior Rating Inventory of Executive Function (BRIEF; parent version) [50,51], and (3) the subscale Cooperation of the Social Skills Rating System (SSRS; parent version) [52,53]. The time management questionnaire gave a more detailed insight into children’s behavior strategies used to improve their time management skills compared to other existing questionnaires (primarily focusing on time perception and/or coordination) and demonstrated good reliability (α=.85) in a pilot study (KCMB, unpublished data, 2016). Secondary outcomes consisted of parent, teacher, and self-reports. Parents filled in the subscale Working Memory of the BRIEF (parent version); the subscales Responsibility, Assertiveness, Self-Control, and Total of the SSRS (parent version); and the It’s About Time Questionnaire (IATQ; parent version) [54]. In addition, teachers were asked to fill in the time management questionnaire, the subscales Plan/Organize and Working Memory of the BRIEF (teacher version), and the SSRS (teacher version) to provide an indication of how the participant functioned at school. Further, we asked participants to fill in a self-efficacy questionnaire (Multimedia Appendix 3) [55]. After receiving the serious game, both parents and participants filled in a satisfaction questionnaire indicating general satisfaction with the serious game on a 10-point Likert scale. Table 1 includes a description of each measure.

**Table 1:** Description of primary and secondary outcome measures.

Measures^a^		Respondent	Description	Cronbach alpha^b^
**Primary outcomes**				
	Time management questionnaire	Parent and teacher report	This 11-item scale is a measure of children’s time management behavior. Parents were asked to rate this behavior on a 10-point Likert scale (ranging from true to not true). The total score ranges from 11 to 110. Higher scores indicate better time management skills.	.83/.90
	BRIEF (subscale Plan/Organize)	Parent and teacher report^c^	A measure of executive functioning in home situations in children aged 5-18 years. For this study, the subscale Plan/Organize, consisting of 12 items, was used to measure children’s planning and organizing skills. The answers are scored on a 3-point Likert scale (never—sometimes—often). The total score ranges from 12 to 36. Higher scores indicate better planning skills.	.81/.80
	SSRS (subscale Cooperation)	Parent and teacher report^d^	A measure of social functioning in children aged 8- 12 years. This questionnaire consists of 4 subscales (ie, Cooperation, Responsibility, Assertiveness, Self-Control) of 10 items each. The answers are scored on a 3-point Likert scale (never—sometimes—often). Two items load on 2 subscales; therefore, the total scale consists of 38 items and has a possible range from 0 to 80. Higher scores indicate better social skills.	.70/.84
**Secondary outcomes**				
	IATQ	Parent report	A measure of children’s skills in time perception and organization. It consists of 25 items scored on a 3-point Likert scale ranging from 0 “rarely” to 3 “almost always.” The total score ranges from 0 to 75. Higher scores indicate better time-oriented behavior.	.74
	Self-efficacy	Self-report	A measure of one’s confidence in his/her ability to carry out specific behaviors related to time management, planning, and social functioning. This measure was constructed in accordance with the standard method for designing self-efficacy scales [55]. As such, it was designed specifically for this study to assess self-efficacy beliefs targeted in the game. Children were asked to rate 14 items on a scale from 0 to 10 how certain they are that they can master certain skills. The total score ranges from 0 to 140. Higher scores indicate more perceived self-efficacy.	.88
	Satisfaction	Parent and self-report	Satisfaction was indicated on a 10-point Likert scale in which both children and parents were asked: “What grade would you give to this game?”	N/A

^a^ BRIEF: Behavior Rating Inventory of Executive Functioning; SSRS: Social Skills Rating System; IATQ: It’s About Time Questionnaire.

^b^ Cronbach alpha is an indication of construct validity. Coefficients were calculated from baseline data in this sample.

^c^ The subscale Working Memory (10 items) from the BRIEF was used as a secondary measure for parents (Cronbach alpha=.83) and teachers (Cronbach alpha=.85).

^d^ The subscales Assertiveness, Responsibility, and Self-Control and the Total Score were used as secondary outcome measures for parents and teachers (except for the subscale Responsibility).

### Statistical Power and Analyses

The sample size was determined in advance by power calculations on the basis of previous pilot study descriptive results (mean, SD) on primary outcome measures, which indicated that 78 participants per group would give 87% power to detect differences of a medium effect size (at least 0.5) between groups (α=.05; 2-sided). In the current study, differences in baseline characteristics were tested with an independent samples *t* test or a chi-square test. For primary and secondary outcome measures, changes from baseline to 10 weeks (reflected by its difference scores) were compared between group 1 and group 2 with ANCOVAs, with baseline score as a covariate and gender and site as factors. To assess improvement during treatment within both groups, paired samples *t* tests were performed on primary and secondary outcome measures before and after playing the serious game. To assess whether effects were maintained after playing the game for 10 weeks within group 1, within-group comparisons of changes at 10 weeks versus 10-week follow-up were performed. Intention-to-treat analyses were used and included all randomized participants. Linear trend at point was used as an imputation method. All statistical analyses were performed using SPSS version 19.0 statistical software (IBM Corp, Armonk, NY, USA) and were 2-sided with a level of significance of α=.05. The significance level for primary outcome measures was adjusted on the basis of the Hochberg procedure [56]. Effect sizes were reported for all analyses using Cohen’s *d* [57].

## Results

### Patient Flow

A total of 170 participants met the inclusion criteria and participated in the study. Mean scores for primary and secondary outcome measures and characteristics of groups 1 and 2 did not differ significantly at baseline (see Table 2). Most participants (91.8%, 156/170) received medication as their treatment as usual. Medication use did not differ between 4 outpatient mental health care clinics and institutions (χ^2^_3_=3.7, *P=*.29).

**Table 2:** Demographic information of the sample at baseline.

Baseline characteristics		Total (N*=*170)	Group 1 (n*=*88)	Group 2 (n*=*82)	Group comparison		
					*t*_168_	χ^2^ (*df*)	*P*
**Sex, n (%)**						0.1 (1)	.72
	Male	137 (80.6)	70 (79.5)	67 (81.7)			
	Female	33 (19.4)	18 (20.5)	15 (18.3)			
Age (years), mean (SD)		9.85 (1.26)	9.89 (1.28)	9.82 (1.24)	–0.36		.79
Total IQ,^a^ mean (SD)		106.18 (14.79)	105.40 (14.46)	107.02 (15.18)	0.72		.55
**ADHD subtypes, n (%)**						3.2 (2)	.21
	Combined	126 (74.1)	66 (75.0)	60 (73.2)			
	Inattentive	38 (22.4)	17 (19.3)	21 (25.6)			
	Hyperactive-Impulsive	6 (3.5)	5 (5.7)	1 (1.2)			
**Attention deficit,** ^b^ **n (%)**						1.6 (1)	.21
	Normal	62 (36.5)	36 (40.9)	26 (31.7)			
	(Sub)clinical	108 (63.5)	52 (59.1)	56 (68.3)			
**Hyperactivity,** ^b^ **n (%)**						1.9 (1)	.17
	Normal	84 (49.4)	39 (44.3)	45 (54.9)			
	(Sub)clinical	86 (50.6)	49 (55.7)	37 (45.1)			
**Oppositional defiant disorder,** ^b^ **n (%)**						2.1 (1)	.14
	Normal	149 (87.6)	74 (84.1)	75 (91.5)			
	(Sub)clinical	21 (12.4)	14 (15.9)	7 (8.5)			
**Game experience, n (%)**						4.3 (3)	.23
	Starter	29 (17.1)	13 (14.7)	16 (19.5)			
	Amateur	55 (32.4)	29 (33.0)	26 (31.7)			
	Experienced	82 (48.2)	42 (47.7)	40 (48.8)			
	Expert	4 (2.4)	4 (4.5)	0 (0)			
Special education? (yes), n (%)		25 (14.7)	14 (15.9)	11 (13.4)		0.2 (1)	.65
Medication use? (yes), n (%)		156 (91.8)	80 (90.9)	76 (92.7)		0.2 (1)	.67
Psychoeducation for parents? (yes), n (%)		9 (5.3)	5 (5.7)	4 (4.9)		0.1 (1)	.82

^a^ IQ: Intelligence Quotient.

^b^ ADHD and ODD severity are based on clinical and subclinical scores on the parent version of the DBDRS.

**Figure 4:**
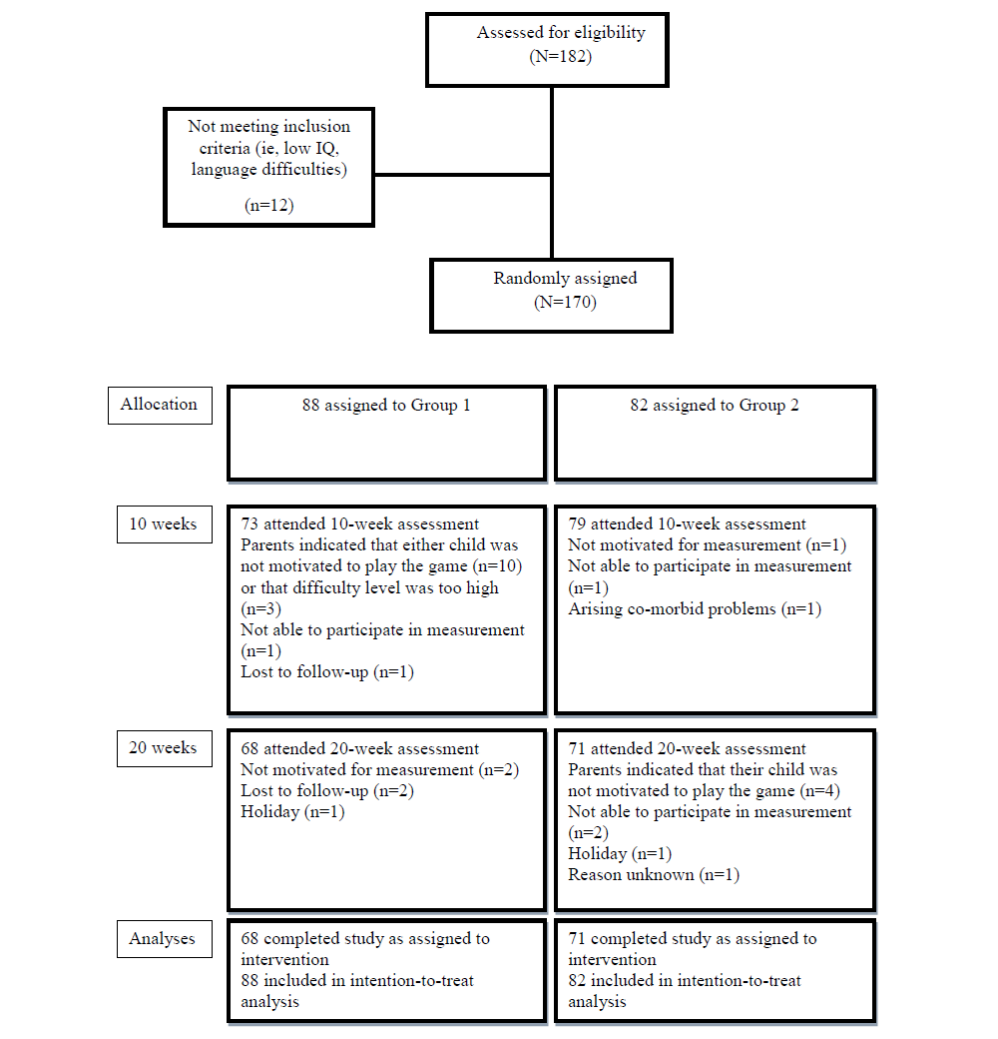
Study flow diagram.

At 10 weeks (T1), 152 of 170 participants (89.4%) completed the study and 139 of 170 (81.8%) completed at the 10-week follow-up (T2). At 10 weeks (T1), the dropout rate was higher in group 1 compared to group 2 (χ^2^_1_=8.0, *P=*.01). The dropout rate did not differ between the 2 study groups at 10-week follow-up (χ^2^_1_=2.5, *P=*.12). Participants who dropped out during the study period did not differ according to age (*t*_168_ *=*–1.34, *P=*.18), gender (χ^2^_1_=2.2, *P=*.13), ADHD subtype (χ^2^_2_=2.5, *P=*.29), or intelligence (*t*_168_ *=–*1.66, *P=*.10) compared to participants who did not drop out (for flow diagram see Figure 4). However, children who dropped out during the study (mean 45.06, SD 15.79) had higher ADHD severity scores compared to children who completed the study (mean 39.07, SD 14.16; *t*_168_=2.09, *P*=.04).

Participants played for a mean 19.04 (SD 9.61) days in the mission-guided game and a mean 11.20 (SD 8.55) days in the closed social community. Additionally, participants played the mission-guided game for a total duration of a mean12.56 (SD 6.57) hours and engaged with the closed social community for a mean 54.27 (SD 70.00) minutes. A difference was seen between group 1 (mean 13.53, SD 6.25) and group 2 (mean 11.53, SD 7.25) with regard to the amount of time playing the mission-guided game (*t*_155_=1.81, *P*=.07) but it did not meet statistical significance. There was a significant difference between group 1 (mean 12.61, SD 8.60) and group 2 (mean 9.70, SD 8.28) with regard to the number of days they engaged with the closed social community (*t*_157_=2.17, *P*=.03). With regard to the amount of time playing in the closed social community, there was a difference between group 1 (mean 1.04, SD 1.16) and group 2 (mean 0.44, SD 1.02; *t*_156_=1.82, *P*=.07), although this was not statistically significant. There were no differences between the 2 groups with regard to the number of days playing the mission-guided game. Both parents (mean 6.96, SD 1.40) and participants (mean 7.33, SD 1.87) reported moderate to high satisfaction with receiving the serious game intervention.

### Between-Group Differences (Group 1 Versus Group 2)

To test the hypothesis that participants playing the serious game would improve on primary and secondary outcome measures, differences between group 1 and group 2 from baseline to 10 weeks (T1; posttest) were evaluated with ANCOVAs (see Table 3). On the primary outcome measures, group 1 showed significantly greater improvements in parent-rated time management skills compared to participants in group 2. Group 1 also showed more improvement in parent-reported planning/organizing skills compared to group 2, although this did not meet statistical significance (*P*=.07). There were no differences concerning participants’ cooperation skills.

**Table 3:** Univariate analyses of covariance comparing group 1 and group 2 on primary and secondary outcome measures during first 10 weeks.

Measures^a^			Group 1 (n=88)		Group 2 (n=82)		ANCOVA		
			Least square mean (SE)	95% CI	Least square mean (SE)	95% CI	*F*_1,163_^b^	*P*	Cohen’s *d*
**Parent-reported**									
	**Primary outcomes**								
		Time management	10.66 (1.64)	7.42, 13.89	4.68 (1.72)	1.29, 8.07	8.56	.004^c^	0.39
		BRIEF (subscale Plan/Organize)	1.47 (0.36)	0.75, 2.18	0.64 (0.38)	–0.11, 1.39	3.32	.07^c^	0.35
		SSRS (subscale Cooperation)	1.10 (0.34)	0.43, 1.78	0.46 (0.36)	–0.25, 1.16	2.32	.13^c^	0.16
	**Secondary outcomes**								
		It’s about time	2.74 (0.73)	1.30, 4.17	1.18 (0.76)	–0.32, 2.68	2.98	.09	0.20
		BRIEF (subscale Working Memory)	0.75 (0.32)	0.11, 1.38	–0.17 (0.33)	–0.83, 0.49	5.16	.02	0.51
		SSRS (Total)	2.24 (0.81)	0.64, 3.83	0.58 (0.85)	–1.09, 2.26	2.68	.10	0.05
		SSRS (subscale Assertiveness)	0.32 (0.27)	–0.22, 0.85	–0.06 (0.28)	–0.62, 0.49	1.28	.26	0.04
		SSRS (subscale Responsibility)	0.75 (0.25)	0.27, 1.23	0.11 (0.26)	–0.39, 0.62	4.28	.04	0.04
		SSRS (subscale Self-Control)	0.24 (0.29)	–0.34, 0.81	0.22 (0.31)	–0.38, 0.83	0	.97	0.07
**Teacher-reported**									
	Time management		5.30 (1.32)	2.70, 7.90	–0.16 (1.38)	–2.88, 2.56	11.05	.001	0.41
	BRIEF (subscale Plan/Organize)		0.78 (0.34)	0.11, 1.44	0.14 (0.35)	–0.55, 0.84	2.30	.13	0.18
	BRIEF (subscale Working Memory)		1.32 (0.34)	0.65, 2.00	0.50 (0.36)	–0.20, 1.20	3.79	.05	0.22
	SSRS (Total)		2.95 (0.67)	1.64, 4.27	2.36 (0.70)	0.98, 3.74	0.51	.48	0
**Self-reported**									
	Self-efficacy		3.06 (2.42)	–0.73, 7.84	–2.13 (2.55)	–7.16, 2.90	2.95	.09	0.26

^a^ BRIEF: Behavior Rating Inventory of Executive Function; SSRS: Social Skills Rating Scale.

^b^ Pillai’s Trace.

^c^ Adjusted *P* values are .01, .14, and .13 for parent-reported time management, BRIEF (subscale Plan/Organize), and SSRS (subscale Cooperation), respectively.

Regarding the secondary outcome measures, group 1 also improved significantly more than the group 2 on measures of parent-reported working memory and responsibility skills. Participants in group 1 showed greater improvements in participants’ time perception compared to group 2, although this did not meet statistical significance (*P*=.09). Teachers reported greater improvements in group 1 than group 2 on the measure of time management and working memory, although this latter effect did not meet statistical significance (*P*=.05). Finally, the same accounted for participants’ self-efficacy in which participants in group 1 showed greater improvements as compared to group 2 (*P*=.09), but it did not meet statistical significance. No differences were found on parent-rated total social skills (with subscales assertiveness and self-control) and teacher-rated total social skills and planning/organizing skills.

### Group 2 Within-Group Effects

Within-group differences for group 2 were evaluated (see Table 4). While receiving treatment as usual for the first 10 weeks, participants improved significantly on parent-reported time management and teacher-reported social skills. After crossing over to the serious game intervention in addition to treatment as usual for the subsequent 10 weeks, significant improvements in outcomes of parent-reported time management, time perception, planning/organizing, working memory, and social skills (primarily cooperation and assertiveness) were found. Furthermore, significant improvements were demonstrated for all teacher-reported outcomes. Self-reported self-efficacy also significantly improved after receiving the intervention (see Table 4).

**Table 4:** Group 2 results of paired samples *t* tests of primary and secondary outcome measures at baseline, 10-week, and 20-week assessments.

Outcomes			Assessment, mean (SD)			T0 vs T1			T1 vs T2		
			Baseline (T0)	10 weeks (T1)	20 weeks (T2)	*t*_81_	*P*	Cohen’s *d*	*t*_81_	*P*	Cohen’s *d*
**Parent-reported** (n=82)											
	**Primary outcomes**										
		Time management	48.88 (15.25)	52.95 (18.17)	60.00 (14.71)	2.80	.006	0.24	4.36	<.001	0.43
		BRIEF (subscale Plan/Organize)^a^	20.41 (4.61)	20.76 (4.54)	22.01 (4.27)	1.07	.29	0.08	3.29	.001	0.28
		SSRS (Cooperation)	8.73 (3.68)	8.90 (3.46)	9.86 (3.16)	0.55	.58	0.05	2.85	.006	0.29
	**Secondary outcomes**										
		It’s about time	30.88 (7.82)	31.61 (7.58)	33.89 (7.15)	1.05	.30	0.09	3.05	.003	0.31
		BRIEF (subscale Working Memory)	14.23 (3.29)	14.42 (3.13)	16.39 (3.36)	0.63	.53	0.06	5.36	<.001	0.61
		SSRS (subscale Assertiveness)	14.52 (3.81)	14.35 (3.73)	15.18 (2.65)	–0.67	.50	0.05	2.91	.005	0.26
		SSRS (subscale Responsibility)	13.63 (3.16)	13.41 (2.92)	13.97 (2.61)	–0.95	.35	0.07	1.97	.05	0.20
		SSRS (subscale Self-Control)	10.06 (3.78)	10.19 (3.95)	10.74 (3.15)	0.50	.62	0.03	1.50	.14	0.20
		SSRS (subscale Total)	44.24 (10.50)	44.08 (10.67)	46.83 (8.84)	–0.23	.82	0.02	2.96	.004	0.28
**Teacher-reported** (n=82)											
	Time management		65.04 (16.37)	64.68 (14.78)	70.20 (10.46)	–0.27	.79	0.02	4.09	<.001	0.43
	BRIEF (subscale Plan/Organize)		20.17 (3.96)	20.16 (3.77)	20.92 (3.18)	–0.05	.96	0	2.40	.02	0.22
	BRIEF (subscale Working Memory)		18.57 (3.73)	18.97 (3.87)	20.42 (3.18)	1.19	.24	0.11	4.11	<.001	0.41
	SSRS (Total)		34.87 (7.62)	36.76 (7.21)	38.37 (6.33)	2.74	.01	0.25	2.52	.01	0.24
**Self-reported** (n=82)											
	Self-efficacy		87.35 (23.63)	86.12 (25.55)	90.87 (22.32)	–0.48	.64	0.05	2.08	.04	0.20

^a^ BRIEF: Behavior Rating Inventory of Executive Function; SSRS: Social Skills Rating Scale.

### Group 1 Within-Group Effects and 10-Week (T2) Follow-Up Effects

Within-group differences for group 1 were then evaluated (see Table 5). While playing the serious game intervention in addition to treatment as usual for the first 10 weeks, significant improvements in outcomes of parent-reported time management, time perception, planning/organizing, and social (primarily cooperation and responsibility) skills were found. Furthermore, significant improvements were demonstrated for participants’ time management, working memory, and social skills as reported by their teachers. Within-group effects showed significant improvement from 10 weeks to 10-week follow-up for parent-reported time management, working memory, time perception, and social skills (primarily cooperation, responsibility, and self-control). Furthermore, significant improvements were demonstrated for teacher-reported time management and working memory skills (see Table 5). This implies that most effects maintained or even further improved at 10-week follow-up.

**Table 5:** Group 1 results of paired samples *t* tests of primary and secondary outcome measures during baseline, 10-week, and 20-week assessments.

Outcomes			Assessment, mean (SD)			T0 vs T1			T1 vs T2		
			Baseline (T0)	10 weeks (T1)	20 weeks (T2)	*t*_87_	*P*	Cohen’s *d*	*t*_87_	*P*	Cohen’s *d*
**Parent-reported** (n=88)											
	**Primary outcomes**										
		Time management	49.73 (16.41)	59.45 (15.28)	64.70 (11.32)	5.82	<.001	0.61	4.66	<.001	0.39
		BRIEF (subscale Plan/Organize)^a^	21.32 (4.21)	22.19 (3.70)	22.58 (3.63)	2.18	.03	0.22	1.25	.22	0.11
		SSRS (Cooperation)	8.53 (2.71)	9.45 (3.24)	10.29 (2.27)	2.62	.01	0.31	3.12	<.01	0.30
	**Secondary outcomes**										
		It’s about time	30.62 (7.21)	33.04 (6.55)	35.08 (6.36)	3.02	.003	0.35	3.48	.001	0.32
		BRIEF (subscale Working Memory)	15.50 (3.52)	16.06 (3.32)	16.78 (3.48)	1.61	.11	0.16	2.29	.03	0.21
		SSRS (subscale Assertiveness)	14.14 (3.33)	14.48 (2.69)	14.63 (3.04)	1.24	.22	0.11	0.62	.54	0.05
		SSRS (subscale Responsibility)	12.83 (2.88)	13.53 (2.69)	14.06 (2.54)	2.83	.006	0.25	2.55	.01	0.20
		SSRS (subscale Self-Control)	9.66 (3.51)	9.93 (3.03)	10.82 (3.05)	0.94	.35	0.08	3.58	.001	0.29
		SSRS (subscale Total)	42.57 (8.81)	44.58 (8.50)	46.85 (8.69)	2.46	.02	0.23	3.93	<.001	0.26
**Teacher-reported** (n=88)											
	Time management		65.31 (16.12)	70.31 (12.43)	73.92 (10.07)	3.45	.001	0.35	2.95	.004	0.32
	BRIEF (subscale Plan/Organize)		20.30 (3.81)	20.87 (2.97)	21.38 (2.39)	1.54	.13	0.17	1.79	.08	0.19
	BRIEF (subscale Working Memory)		18.49 (3.65)	19.75 (3.33)	20.62 (2.47)	3.87	<.001	0.36	2.55	.01	0.30
	SSRS (Total)		33.73 (9.42)	36.75 (6.92)	36.38 (7.04)	4.03	<.001	0.37	–0.52	.60	–0.05
**Self-reported** (n=88)											
	Self-efficacy		89.39 (25.03)	92.33 (22.01)	94.09 (20.66)	1.29	.20	0.12	1.62	.29	0.08

^a^ BRIEF: Behavior Rating Inventory of Executive Function; SSRS: Social Skills Rating Scale.

### Adverse Events

While playing the serious game, adverse events were registered by the researcher and checked by a health care professional. Overall, there were 10 adverse events that could be related to the intervention that were reported by parents, teachers, or participants themselves. All adverse events were of mild (n=5) or moderate (n*=*5) severity, but this was no reason to discontinue study participation. Examples of adverse events were pain in the fingers, irritability, and headache. An adverse event was a reason to discontinue the study for only one known participant. This participant did not want to play the game anymore because he could not concentrate during his school activities. Sounds reminded him of the game and this consequently distracted and frustrated him. No serious adverse events were reported.

## Discussion

The findings of this 20-week multisite randomized controlled crossover open-label trial demonstrate the efficacy of an Internet-based serious game specifically developed for children with ADHD. Participants who played the serious game during the first 10 weeks significantly improved in their daily life functioning across domains of time management, social skills (eg, responsibility) and working memory compared to participants in group 2. These effects were small to medium and were maintained or even further improved at the 10-week follow-up for group 1. Children from group 2, who played the serious game during the second period of the study (weeks 10 to 20), improved on comparable domains of daily life functioning over time. In contrast to previous studies that typically demonstrate that computerized neurocognitive interventions for ADHD improve working memory skills but do not have a strong impact on daily life functioning (“far-transfer effects”) [26-30,58], the findings of the current study provide clear evidence that a serious game for children with ADHD can improve the performance of these children in important daily life skills.

Of particular interest is the clear effect seen on time management skills because dysfunctional time management is one of the core problems in ADHD, affecting social and executive domains of daily life functioning [4,37]. It should be noted that the improvements in time management and working memory were reported by parents at home and teachers at school supporting the claim that positive behavioral adaptations resulting from use of the serious game generalized across different settings. Although improvements in planning/organizing skills have been shown by other computerized neurocognitive training programs as well [39,59], this serious game is unique because it elicits its effects by promoting behavioral strategies instead of training executive functions by offering repeated cognitive exercises. As such, this approach provides sustainable therapeutic effects by improving behavioral strategies that can be applied in daily life.

Plan-It Commander demonstrated improvement of total social skills over time, but had nonsignificant between-group effects as reported by parents and teachers. Multiplayer and cooperative game play could be more explicitly integrated to improve social benefits of the current game format. Improvements in social responsibility among players was observed. This was expected given that game elements, such as a mentor figure or nonplayable characters and peers with ADHD with whom they could interact (eg, asking for help, being polite, and dealing with compliments in a good way), enabled players to practice socially responsible behaviors in the game that could be practiced in the “real world” as well. This finding is important given that well-developed social responsibility skills in children contribute to academic success and an optimal learning environment [52,53].

Another goal of Plan-It Commander was to improve children’s self-efficacy. Children were more confident in self-control with regard to their time management and planning skills and engagement in positive social interactions. However, the between-group effect on self-efficacy did not meet statistical significance. It may be important that further development of serious gaming addresses aspects of the concept of self-efficacy (eg, modeling behavior) more thoroughly because increased self-efficacy has been shown to correlate significantly with self-esteem and adaptive behaviors such as persistence in reaching goals in daily life [60-62]. Overall, this study introduces serious gaming as an effective and attractive behavioral intervention for children with ADHD, especially for time management with evidence for effects on certain social skills and self-efficacy as well.

### Clinical Implications

ADHD is a chronic health problem and previous studies have emphasized the need for efforts to treat impairments outside the therapy context and provide patients with greater autonomy [22,23,63]. The Internet-based serious game intervention in this study fulfills this need by addressing impairments associated with ADHD among school-aged children in the home and school context. Results demonstrated that parents as well as children were satisfied with their treatment. The current intervention was positioned as an adjunct to treatment as usual. No therapist or parent explicitly intervened during the game intervention. Furthermore, no additional rewards were given and no prompts to play the game regularly were explicitly provided outside the game. Given that young patients with ADHD have engagement and motivation issues in general, easy accessible interventions such as serious games can stimulate them to comanage their health care processes as part of the Chronic Care Model of Child Health and the World Health Organization Mental Health Action Plan 2013-2020 [22,23,64].

The current intervention is unique in its contribution to the adjunctive ADHD treatment repertoire because it differs from existing computerized neurocognitive training formats. Instead of requiring the repetition of executive function tasks normally presented in neurocognitive training format for children with ADHD, Plan-It Commander offers behavioral strategies (e.g., reinforcement, immediate performance feedback from a mentor, goal setting through missions, modeling, social support, and comparison) that increase functional outcomes within a relatively short period of time. Even more important is the fact that participants labeled as “clinically stable” by their clinicians still showed significant improvements in daily functioning. It is encouraging that significant results were obtained over and above medication effects. Future research could examine the effects of this serious game in a nonmedicated sample to disentangle its effects. Notably, participants with higher severity scores on ADHD symptoms were more likely to drop out from the study, which implies that we can only generalize our results to children with less severe ADHD symptoms, but this remains speculative because symptoms were within the normal range. Furthermore, future research should consider family factors (eg, social support network, socioeconomic status, parental ADHD) as well in contributing to study dropout.

### Limitations

The results of this study must be considered in the light of several limitations. Group 2 followed treatment as usual and did not use a nontherapeutic “placebo game.” Therefore, this study could be controlled for changes in time and effects of repeated measurements, but not for placebo effects. Further, parents were not rater-blinded and rater-blindness of teachers could not fully be guaranteed because children were free to report game experiences. Questionnaires to assess time management and self-efficacy were designed on theoretical basis and guidelines by Bandura [55]. Both instruments show good reliability. The time management questionnaire was developed because of a lack of instruments for this age group. This questionnaire was used previously in a randomized controlled pilot study (KCMB, unpublished data, 2016). Future research should evaluate the psychometric characteristics of these questionnaires in more detail.

### Conclusions

The current randomized controlled study demonstrated that Plan-It Commander is an effective adjunctive Internet-based behavioral intervention for children with ADHD. It is a unique contribution to the literature on serious games because it showed that a serious game for ADHD, as an adjunct to treatment as usual, improves functional outcomes of time management as well as working memory and social responsibility. It fits the current interest in nonmedical treatment options for ADHD and stimulates young children to manage their impairments by offering an easy, accessible home treatment intervention. The findings contribute to scientific knowledge about the impact of serious game interventions on behavioral outcomes, Internet-based interventions for mental health that are consistent with the Chronic Care Model of Health, and innovative approaches to treating people coping with chronic mental health conditions.

## Multimedia Appendix 1

CONSORT-EHEALTH checklist V1.6.2 [65].

## Multimedia Appendix 2

Time management questionnaire.

## Multimedia Appendix 3

Self-efficacy questionnaire.

## Abbreviations

ADHD: attention-deficit/hyperactivity disorder
BRIEF: Behavior Rating Inventory of Executive Functioning
DBDRS: Disruptive Behavior Disorders Rating Scale
IATQ: It’s About Time Questionnaire
IQ: intelligence quotient
K-SADS: Kiddie Schedule for Affective Disorders and Schizophrenia-Lifetime
SSRS: Social Skills Rating System
WISC-III: Wechsler Intelligence Scale for Children III
