# Supplementary material for: Behavioral Outcome Effects of Serious Gaming as an Adjunct to Treatment for Children With Attention-Deficit/Hyperactivity Disorder: A Randomized Controlled Trial
Source: J Med Internet Res. 2016 Feb 16;18(2):e26. doi: 10.2196/jmir.5173 (PMC4773597; doi:10.2196/jmir.5173)
Supplement: Supplementary file 2 [file jmir_v18i2e26_app2.pdf]

Appendix 2. Time management questionnaire.

Instructions

Please read the statements below and respond to each one by circling the number (from 1 to 10) which best describes your child during the past two weeks.

False ... True

	1	2	3	4	5	6	7	8	9	10
1. My child is able to finish a task within a pre-defined time frame.										
2. My child can figure out how much time is needed to finish a task before the deadline.										
3. My child is able to perform the morning routine by himself/herself within a certain time frame.										
4. My child is able to independently perform daily activities within a reasonable time frame.										
5. My child is able to adjust his/her homework planning on the basis of other activities (for example: starting earlier to anticipate going to sport or music class that evening)										
6. My child is able to start his/her long-term projects on time thereby minimizing the chance of time pressure.										
7. My child often finishes his/her homework before going to bed.										

8. My child makes good decisions about setting priorities when time is restricted (for example: going home immediately after school to finish a long-term assignment instead of playing with friends).	○	○	○	○	○	○	○	○	○	○
9. My child is able to work on a long-term project over several days.	○	○	○	○	○	○	○	○	○	○
10. My child bungles his/her task at the last moment.	○	○	○	○	○	○	○	○	○	○
11. My child regularly checks his/her watch to keep track of the time.	○	○	○	○	○	○	○	○	○	○
