# Supplementary material for: Behavioral Outcome Effects of Serious Gaming as an Adjunct to Treatment for Children With Attention-Deficit/Hyperactivity Disorder: A Randomized Controlled Trial
Source: J Med Internet Res. 2016 Feb 16;18(2):e26. doi: 10.2196/jmir.5173 (PMC4773597; doi:10.2196/jmir.5173)

Appendix 3. Self-efficacy questionnaire.

Instructions

Fill in the example first, so as to know how to answer the real questions below.

Example

If you were asked to lift up several materials with a certain weight, how certain are you that you can lift up the following items? Indicate how certain you are by choosing a number from 0 to 10 on the scale below.

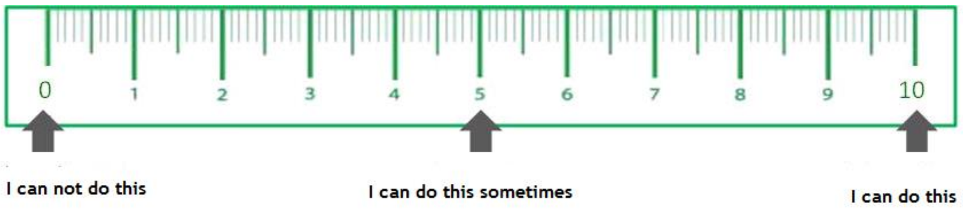

I can lift up a sugar pack

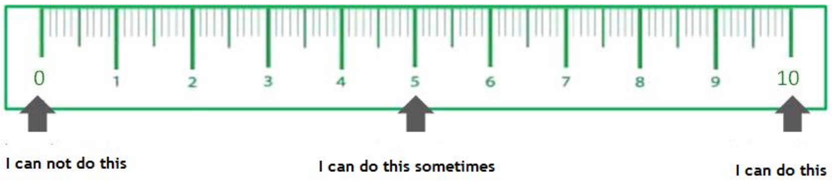

I can lift up a packed suitcase

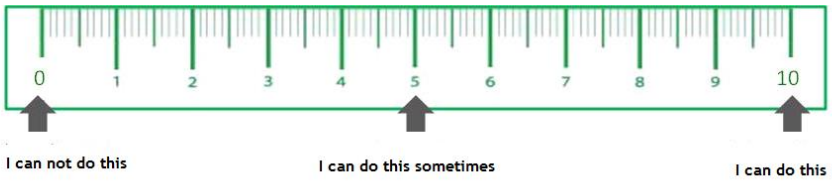

I can lift up a table

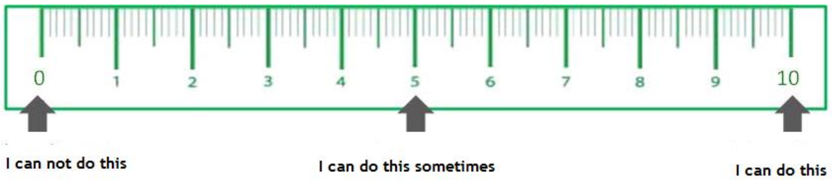

I can lift up a car

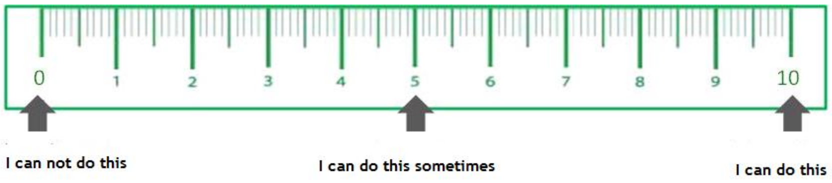

Instructions

We designed this questionnaire to gain a better understanding about the things children have difficulties with.

Indicate below how certain you are that you can do these things by choosing a number from 0 to 10.

1. I am confident that I can listen to instructions while I am in a hurry.

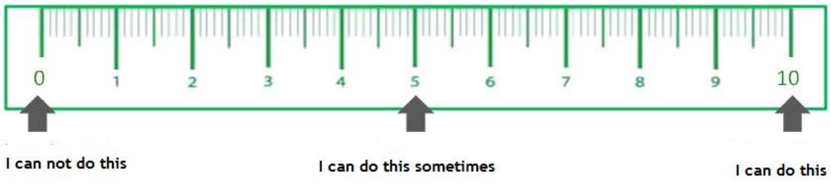

2. I am confident that I can plan my tasks within a certain time frame.

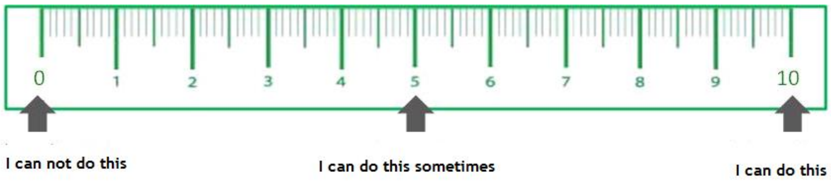

3. I am confident that I can help a friend or classmate, even when I have things to do.

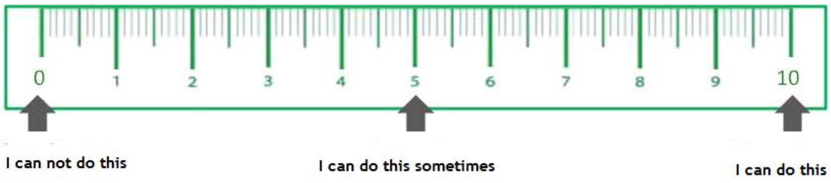

4. I am confident that I can keep track of time by looking at a clock or watch, so that I get things done on time.

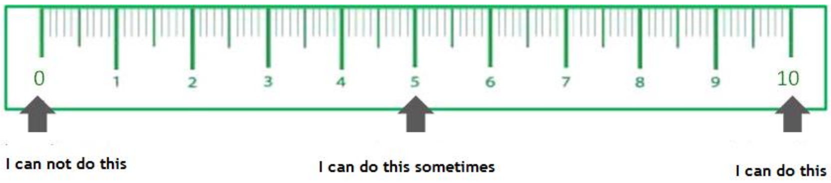

5. I am confident that I can listen to instructions people give me without interrupting them.

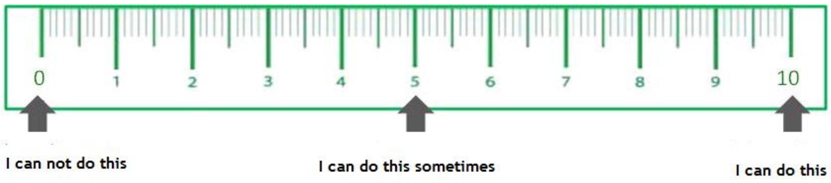

6. I am confident that I can get things done in the amount of time I planned to get them done.

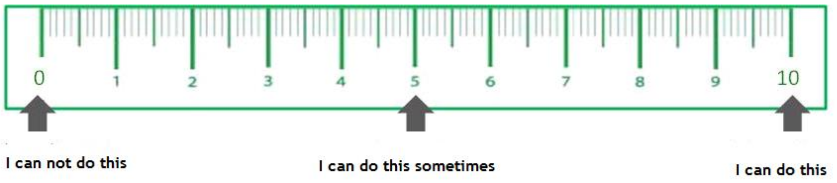

7. I am confident that I can stay focused so that I get things done in the amount of time I planned.

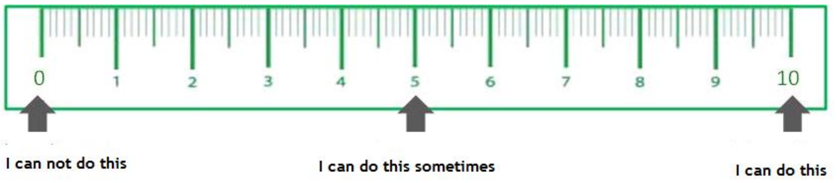

8. I am confident that I can do my tasks within the time I planned.

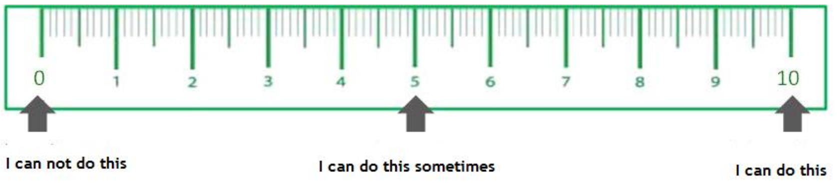

9. I am confident that I can keep trying to make a plan that works even if I fail the first time.

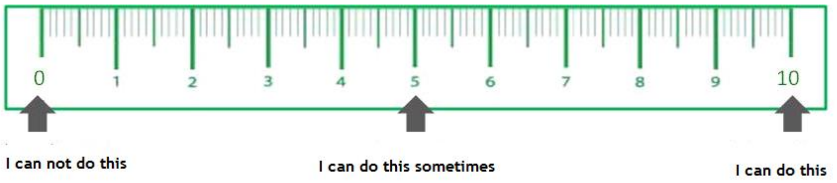

10. I am confident that I can practice to get better at finishing my tasks on time.

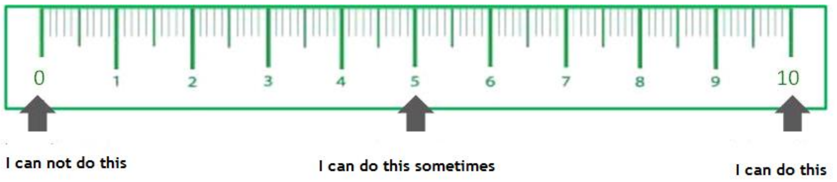

11. I am confident that I can stay calm and keep trying to succeed even when my plan doesn’t work.

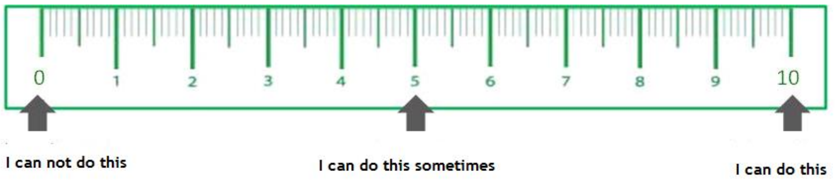

12. I am confident that I can get things I need to get done without getting distracted or bored.

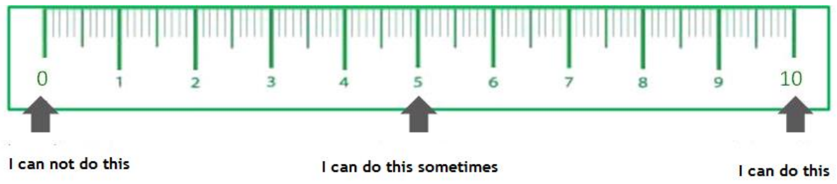

13. I am confident that I can learn new ways to finish my tasks on time.

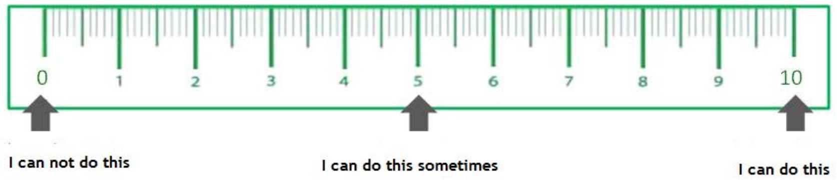

14. I am confident that I can keep trying to make good plans even if my plans don’t work out.

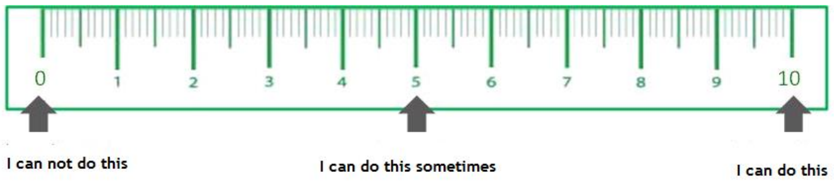

Supplement: Supplementary file 3 [file jmir_v18i2e26_app3.pdf]
